# Sonographic and Clinical Progression of Adenomyosis and Coexisting Endometriosis: Long-Term Insights and Management Perspectives

**DOI:** 10.3390/jpm15110538

**Published:** 2025-11-06

**Authors:** Francesco Giuseppe Martire, Claudia d’Abate, Eugenia Costantini, Maria De Bonis, Giuseppe Sorrenti, Gabriele Centini, Errico Zupi, Lucia Lazzeri

**Affiliations:** 1 Obstetrics and Gynecological Clinic, Department of Molecular and Developmental Medicine, University of Siena, 53100 Siena, Italy; francescogmartire@libero.it (F.G.M.); eugenia.costantini22@gmail.com (E.C.); mariadebonis@gmail.com (M.D.B.); centini.gabriele@gmail.com (G.C.); lucialazzeri79@gmail.com (L.L.); 2 Department of Gynecology, San Carlo di Nancy Hospital, 00165 Rome, Italy; giuseppesorrenti@gmail.com

**Keywords:** adenomyosis, different managements, endometriosis, follow-up, patient-centered care, precision medicine, specific symptoms, tailored therapy

## Abstract

**Objectives:** To evaluate the impact of hormonal therapy on the evolution of painful symptoms in premenopausal women with adenomyosis, with or without concomitant endometriosis, over an 18-month follow-up period. This study aimed to compare the symptomatic progression between treated and untreated patients, highlighting the potential role of medical therapy in symptom control and disease stabilization. Secondary, an objective was to explore sonographic changes within our study population, in parallel with clinical outcomes. **Methods:** This retrospective observational study, conducted at the Endometriosis Referral Center of the University Hospital of Siena, included 40 women with ultrasound evidence of adenomyosis with and without endometriosis. The population was divided into two groups: 20 patients receiving hormone treatment and 20 not receiving hormone treatment. All patients underwent clinical and ultrasound examinations throughout an 18-month follow-up period, during which types, locations, degrees of disease, and associated symptoms were evaluated. **Results:** Forty patients enrolled in the study presenting with symptoms such as dysmenorrhea, dyspareunia, and heavy menstrual bleeding were included. A total of 22 patients showed isolated adenomyosis, while 18 adenomyosis and endometriosis both. The mean age was 38.5 years (±4.2 SD), with 57.5% being nulliparous. The types of adenomyosis detected were focal in 25%, diffuse in 50%, and mixed (both focal and diffuse) in 25%. Disease severity was classified as mild in 15%, moderate in 45%, and severe in 40%. After 18 months of continuous hormonal therapy, a reduction in focal adenomyosis was observed in 10%, and an improvement of dysmenorrhea and Heavy Menstrual Bleeding (HMB) was noted, while a slight ameliorating of dyspareunia was detected. In contrast, untreated patients showed either unchanged or worsened symptoms. **Conclusions:** The findings underscore the value of medical therapy in the management of adenomyosis, supporting current recommendations. Given the heterogeneity of clinical presentations and frequent overlap with endometriosis, a personalized treatment approach remains essential. Further larger-scale, long-term studies are needed to confirm these preliminary results and explore the potential impact on fertility preservation.

## 1. Introduction

Adenomyosis is a benign, long-standing uterine disorder in which endometrial glands and stromal tissue are ectopically located within the myometrium. First systematically described by Cullen in the early 20th century, the disease continues to manifest through a relatively stable set of symptoms, most notably heavy menstrual bleeding (HMB) and various forms of pelvic discomfort, including dysmenorrhea, dyspareunia, and chronic pelvic pain [1].

From a morphological standpoint, adenomyosis is generally classified into three subtypes: diffuse adenomyosis, focal adenomyosis, and adenomyoma, the latter presenting as a nodular lesion [2]. These subtypes differ not only in distribution but also in the depth of tissue infiltration, which may involve the inner (junctional zone) or outer myometrial layers. Severity grading—mild, moderate, or severe—is typically based on ultrasonographic criteria, including lesion size, thickening of the affected uterine wall, and the number of direct and indirect imaging features present. For the purposes of this study, a schematic scoring system was applied, considering both the number and the extent of sonographic criteria identified [2,3,4]. However, no consensus currently exists within the MUSA group regarding a standardized grading of disease severity.

In some cases, disease morphology is associated with symptom severity. For example, extensive diffuse adenomyosis has been linked to more intense dysmenorrhea and heavier bleeding than focal disease, and diffuse sonographic patterns are more frequently detected in older patients with HMB. Nonetheless, the clinical picture does not always parallel disease extent, as individuals with marked histological involvement may experience milder symptoms than those with limited disease. This inconsistency may partly result from concurrent gynecological conditions such as endometriosis.

Reported prevalence rates for adenomyosis range widely, from 1% to 70%, reflecting variability in diagnostic definitions and the historical reliance on postoperative histopathology [5,6]. Traditionally, the disorder was most often identified in multiparous, premenopausal women in their forties, typically following hysterectomy performed for chronic pelvic pain and/or HMB after the conclusion of childbearing [5,6].

In recent years, the refinement of non-invasive imaging techniques, including high-resolution transvaginal ultrasonography (US) and magnetic resonance imaging (MRI), has facilitated earlier detection and broadened epidemiological data to encompass younger patient groups [7,8]. These findings have led to a paradigm shift, indicating that adenomyosis may develop earlier in life and result from a spectrum of pathogenetic mechanisms extending beyond uterine trauma and hyperperistalsis, potentially involving congenital and molecular factors [9].

Given its capacity to significantly impair quality of life in early-onset cases, prompt, non-invasive diagnosis and tailored therapeutic strategies are essential to limit disease progression, preserve reproductive potential, and improve patient outcomes [10,11].

The present investigation aimed to evaluate the progression of adenomyosis, both with and without coexisting endometriosis, and to assess associated painful symptoms in premenopausal women over an 18-month follow-up period, while also examining the impact of hormonal therapy. The primary objective was to compare symptom patterns between patients with isolated adenomyosis and those presenting with concomitant endometriosis. The secondary objective was to explore changes in symptom severity over time in relation to treatment modality, contrasting outcomes between patients receiving hormonal therapy and those who remained untreated. A further objective was to investigate sonographic changes in the study population, complementing the clinical findings and providing additional insights into disease progression.

## 2. Materials and Methods

This pilot retrospective observational study was conducted at the Endometriosis Referral Center of the University Hospital of Siena, which serves as a recognized hub for the diagnosis and management of endometriosis and adenomyosis. The study included a small cohort of premenopausal women referred for adenomyosis, who underwent evaluation and had at least 18 months of ultrasound follow-up at our unit. The primary objective was to compare symptom profiles between patients with isolated adenomyosis and those with concomitant endometriosis. The secondary objective was to assess changes in symptom severity over time according to therapeutic approach, contrasting patients undergoing hormonal treatment with those managed without pharmacological intervention. An additional key objective was to assess sonographic modifications within the study cohort, integrating these findings with clinical outcomes to yield further understanding of disease progression. This approach reflects the principles of personalized medicine, in which clinical decisions and therapeutic strategies are tailored to the specific characteristics and disease evolution of each patient.

Each time a patient visits our center, clinical information is collected and consent is requested for the processing of personal data for research purposes.

Inclusion criteria comprised premenopausal status; absence of hormonal therapy at the time of the first evaluation; no previous surgical treatment for endometriosis; availability of a complete clinical history; transvaginal sonography (TVS) at baseline and during follow-up; at least 18 months of combined clinical and ultrasound surveillance; signed informed consent for TVS examination; and authorization for personal data analysis.

Exclusion criteria included menopausal status; pregnancy at baseline; incomplete clinical or imaging records; prior pelvic surgery for benign gynecological conditions such as pelvic inflammatory disease, endometriosis, or ovarian cysts; refusal to undergo transvaginal ultrasound; absence of informed consent for data processing; or incomplete medical history and symptom reporting.

All data were collected and stored in a secure hospital database routinely used in our institution for clinical and research purposes.

The study was conducted in accordance with the Declaration of Helsinki and approved by Ethics Committee of the Azienda Ospedaliera-Universitaria Senese, Siena, Italy (protocol code 25532, approved on 16 October 2023). Data collection began after the approval date in October 2023 and was conducted retrospectively. In particular, data collection referred to patients previously seen from November 2022 onward. No new patients were enrolled after the Ethics Committee approval; rather, we continued the follow-up of the same cohort of patients.

In line with our routine clinical practice, for each patient included in the study demographic and anthropometric data were collected, including date of birth, age at ultrasound, body mass index (BMI), age at menarche, parity, menstrual cycle characteristics, date of last menstrual period, and previous surgical interventions. Information on ongoing medications was also obtained, with specific attention to the use of hormonal treatments and non-steroidal anti-inflammatory drugs (NSAIDs) for dysmenorrhea.

Pain-related symptoms associated with adenomyosis and/or endometriosis were assessed in all patients prior to TVS examination. The symptoms evaluated included dysmenorrhea, dyspareunia, dysuria, dyschezia, and chronic pelvic pain (CPP)—the latter defined as persistent discomfort occurring outside menstruation for at least seven days in six or more consecutive months. Symptom severity was quantified using a 10 cm Visual Analog Scale (VAS), where 0 represented “no pain” and 10 corresponded to “maximum pain.” Only symptoms with VAS ≥5 were considered clinically significant, to focus on moderate-to-severe cases more likely to impact quality of life, while milder symptoms (VAS 1–4) were excluded. Menstrual blood loss was assessed through patient self-report a method considered reliable and comparable to the pictorial blood loss analysis chart (PBCA) [12,13].

The US examination was performed using a Voluson S 10 device (GE Healthcare; Zipf, Austria) with a 3D transvaginal sonography probe. All scans were stored as 2D still images, 2D video clips, and 3D volumes. Ultrasound evaluation was performed by two experienced operators (F.G.M. and L.L.) under blinded conditions.

We evaluated the uterus, the adnexa, pouch of Douglas and other pelvic organs (bladder, rectum, rectosigmoid junction, and ureters) and sites (posterior, lateral and anterior parametria, rectovaginal septum, vesicouterine pouch, and uterosacral ligaments [USLs]).

Sonographic uterine adenomyosis findings were recorded according to MUSA criteria [3,4]. These morphological adenomyosis features have been described previously, and there is a broad consensus that they are reliable morphological adenomyosis markers [3,4,14]. Briefly, the adenomyosis diagnosis was made when at least one US direct sign of adenomyosis features was present: myometrial cystic areas, hyperechoic islands, linear striations, and buds on either 2D or 3D imaging [4]. The association of indirect signs, such as irregular/infiltrated endometrial-myometrial junctional zone and others, was also recorded.

Although there is no international MUSA consensus on the severity of the disease, adenomyosis was classified as mild, moderate, or severe according to our recently published classification scoring system, particularly the severity of the disease was calculated using a schematic scoring system that considers the number and extent of sonographic criteria [2,15]. The main parameters included myometrial thickness and asymmetry, the presence and number of myometrial cysts, irregularity or disruption of the endometrial–myometrial junction, and the extent of myometrial involvement. The classification was defined as mild when only one or two features were present, moderate when several criteria were observed with partial uterine wall involvement, and severe when multiple features coexisted with extensive and diffuse myometrial disease [2,15].

Due to the limited number of cases included in this pilot study, we decided to group together patients with focal adenomyosis and those with adenomyoma under the single category of “focal adenomyosis” for analytical purposes.

Ultrasound images were retrospectively reviewed by expert gynecologists specialized in Level II gynecologic ultrasound, who were blinded to the patients’ clinical history and treatment status at the time of evaluation. Importantly, all ultrasound exams had originally been performed by the same group of experienced gynecologist–sonographers. Our institution is a referral center for endometriosis, ensuring a high level of standardization in both acquisition and interpretation of the images.

The diagnosis of endometriosis was made exclusively by transvaginal ultrasound, in accordance with standardized and validated sonographic criteria. Evaluation for concomitant endometriosis was performed using a previously published ultrasound mapping protocol and IDEA group guidelines [16]. The diagnosis of endometrioma was based on the presence of a persistent unilocular or multilocular (<5 locules) cyst with homogeneous low-level echogenicity and absent or moderate vascularization [17]. Deep endometriosis was diagnosed when hypoechoic linear or nodular retroperitoneal lesions with irregular margins and absent or minimal Doppler signals were visualized in the anterior or posterior pelvic compartments, according to validated criteria [16].

At baseline, none of the patients were receiving medical therapy. In our center, all patients presenting with a clinical condition requiring treatment systematically undergo counseling regarding available therapeutic options; therefore, in this cohort as well, medical treatment was proposed when appropriate, including continuous progestin therapy, combined estrogen-progestin therapy (continuous or cyclic), or other options tailored to the individual patient. Twenty patients initiated continuous progestin therapy with Dienogest 2 mg per day, while twenty declined hormonal treatment. Among the eighteen patients with concomitant endometriosis, nine received hormonal therapy. The cohort composition was intentional, as part of the design of this pilot study aimed at exploring potential differences between the two groups.

All patients who started medical treatment maintained it without interruption throughout the follow-up.

Follow-up evaluations were scheduled every six months for a total of 18 months. At each visit, TVS was performed, adenomyosis and endometriosis lesions were documented, and symptoms were reassessed using the VAS, comparing scores with baseline and prior follow-up assessments. Lesion changes were classified as size reduction, resolution, stability, or appearance of new lesions.

All statistical analyses were conducted using SPSS v.15.0 (SPSS Inc., Chicago, IL, USA). Continuous normally distributed variables are presented as mean ± standard deviation (SD), and non-normally distributed variables as median. Categorical variables are expressed as counts and percentages.

Comparisons between groups (hormonal treatment vs. no hormonal treatment) were performed using Student’s *t*-test for continuous variables and Fisher’s exact test for categorical variables. Statistical significance was defined as *p* < 0.05 for all analyses.

## 3. Results

**Figure 1:**
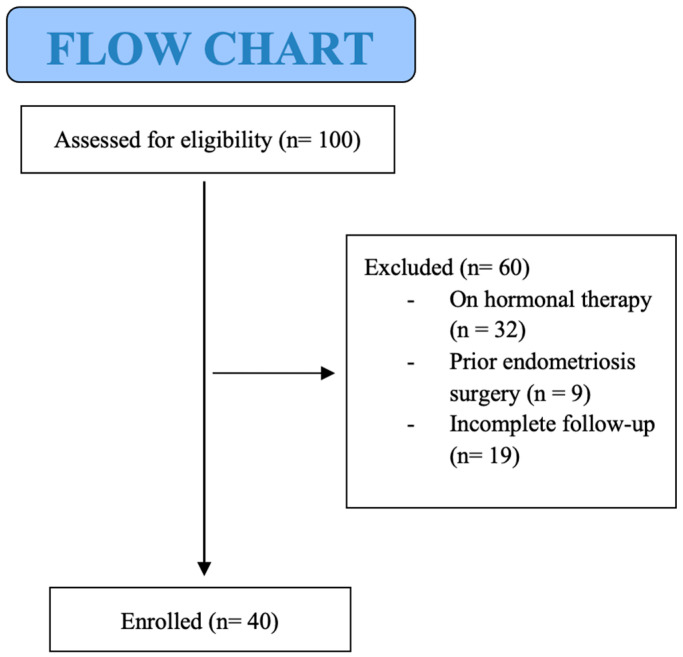
CONSORT diagram.

Among 100 patients of fertile age with suspected adenomyosis, who underwent clinical examination and ultrasound at a dedicated endometriosis and adenomyosis clinic in our Gynecology Unit between November 2022 and January 2025, 60 patients did not meet all inclusion criteria, and 40 patients of fertile age were included in the study (Figure 1).

The characteristics and painful symptoms reported at baseline evaluation for the entire study population and the patients included in the study are presented in Table 1. The mean age of our patients was 38.5 years ± 4.2 SD. The mean BMI was 20.2 ± 2.1 SD, the average age at menarche was 12.6 ± 1.5 SD, the pregnancy rate was 0.8 ± 0.3 SD, 17/40 (42.5%) patients had at least one pregnancy, while 23/40 (57.5%) were nulliparous.

We evaluated all painful symptoms including dysmenorrhea, dyspareunia, dysuria, dyschezia, chronic pelvic pain, and heavy menstrual bleeding (PBAC >100). In particular, we observed that dysmenorrhea was the most commonly reported symptom, followed by HMB.

Among the 40 patients included in the study, dyspareunia was reported by 18 out of 40 (45%) patients, while dysuria was less common with average prevalences of 17.5%. Dyschezia was reported by 8 out of 40 (20%) patients.

Additionally, other symptoms, such as headaches, bowel irregularities, leukorrhea, mood changes, and urinary tract infections, were reported by 9 out of 40 (22.5%) patients.

In addition, in Table 1, patients are classified according to the type of adenomyosis identified during the US evaluation: 10 out of 40 (25%) have focal adenomyosis, 20 out of 40 (50%) have diffuse adenomyosis, and 10 out of 40 (25%) presents both focal and diffuse adenomyosis. Additionally, in 32.5% of cases (13/40 patients), there is involvement of the inner myometrium, 50% (20/40 patients) exhibit involvement of the outer myometrium, and 17.5% (7/40 patients) have both inner and outer myometrial involvement.

The severity of the disease was also assessed, with 15% of patients (6/40) showing mild disease, 45% (18/40) moderate disease, and 40% (16/40) severe disease.

Furthermore, Table 1 explores a potential correlation between adenomyosis and endometriosis, reporting the possibility of concurrent endometriomas (OMA), and deep endometriosis (DE), mostly involvement of the uterosacral ligament (USL). Specifically, at the first ultrasound control (T = 0), 5 patients had endometriomas (12.5%), 4 (10%) had deep infiltrating endometriosis with bowel involvement, 3 (7.5%) involvement of the rectovaginal septum (RVS) and 2 (5%) had bladder involvement. For uterosacral ligament involvement, 13 patients (32.5%) had left-sided USL involvement, and only 3 patients (7.5%) had right-sided involvement.

We analyzed (Table 2) a possible correlation between each symptom and types and the degree of adenomyosis such as focal or diffuse adenomyosis, involvement of the inner or outer myometrium, and intensity of the pathology classified as mild, moderate, or severe.

Specifically, dysmenorrhea was found in 32 patients. Of these, 9 (28.1%) had a diagnosis of focal adenomyosis, 20 (62.5%) had diffuse adenomyosis and 3 (9.4%) had focal and diffuse adenomyosis; 12 patients (37.5%) had inner myometrial involvement, while 18 (56.3%) had outer myometrial involvement and 2 had inner and outer myometrial involvement. Regarding severity, most patients with dysmenorrhea had moderate pathology (46.9%).

Another finding reported by the patients was HMB. Patients reporting this symptom were diagnosed with focal adenomyosis in 33.3% of cases, diffuse adenomyosis in 53.3% of cases, and both types in 13.3% of cases. Among them, most of the patients had inner myometrial involvement. In terms of disease severity in patients with heavy bleeding, 6 patients (20%) had mild pathology, 15 patients (50%) had moderate pathology, and 9 patients (30%) had severe pathology.

Dyspareunia was reported by 18 patients, half patients had diffuse adenomyosis (50%) and most of all the patients (55.6%) had outer myometrial involvement. Again, moderate forms predominated, found in 11 patients (61.1%), followed by severe forms in 5 patients (27.8%) and mild forms in 2 patients (11.1%).

Chronic pelvic pain was found in 16 patients, of whom 9 (56.3%) had diffuse adenomyosis. Among them, 6 patients (37.5%) had inner myometrial involvement, and 8 (50%) had outer myometrial involvement and 2 (12.5%) both. In terms of disease severity, 37.5% of patients had moderate and severe forms. Dysuria and dyschezia were less frequent symptoms and only 9 patients reported symptoms not strictly associated with adenomyosis.

During the follow-up, among the total study population not receiving therapy at baseline, 20 patients with ultrasound evidence of adenomyosis initiated continuous hormonal therapy (progestin without suspension), while 20 patients opted not to undergo hormone therapy.

The data show a progressive reduction in focal adenomyosis among treated patients, with sonographic signs observed in 25% of cases at 6 months, 15% at 12 months, and 10% at 18 months. In contrast, the reduction in diffuse adenomyosis is less pronounced, with ultrasound signs present in 65% of patients at both 6 and 12 months, decreasing slightly to 60% after 18 months. On the other hand, ultrasound findings in patients who did not undergo any treatment over the course of the six-monthly follow-up, show a worsening in disease extent, including the transition from focal to diffuse forms and the progressive involvement of both focal and diffuse adenomyosis. Specifically, the prevalence of the diffuse form increased from 50% (10 out of 20 patients) at 12 months to 60% (12 out of 20) at 18 months. Similarly, combined involvement of both forms rose consistently over time: from 20% of patients at 6 months, to 25% at 12 months, and 30% at 18 months.

Additionally, an improvement in disease severity has been observed. After 6 months of treatment, 40% of patients presented with a moderate form of the disease, decreasing to 30% after 12 months and 25% after 18 months. Similarly, the prevalence of the mild form increased over time, being observed in 5% of patients after 6 months, 15% after 12 months (including those who were previously diagnosed with moderate adenomyosis), and 5% after 18 months. However, no significant ultrasound changes were noted in patients with severe disease. In contrast to patients receiving continuous progestin-based hormone therapy, those in the untreated group experienced a deterioration in adenomyosis severity, with the proportion of severe cases increasing from 35% to 40% over the follow-up period, and moderate cases rising from 40% to 55%. As a consequence, the prevalence of mild forms declined markedly, from 25% to 5%.

**Figure 2:**
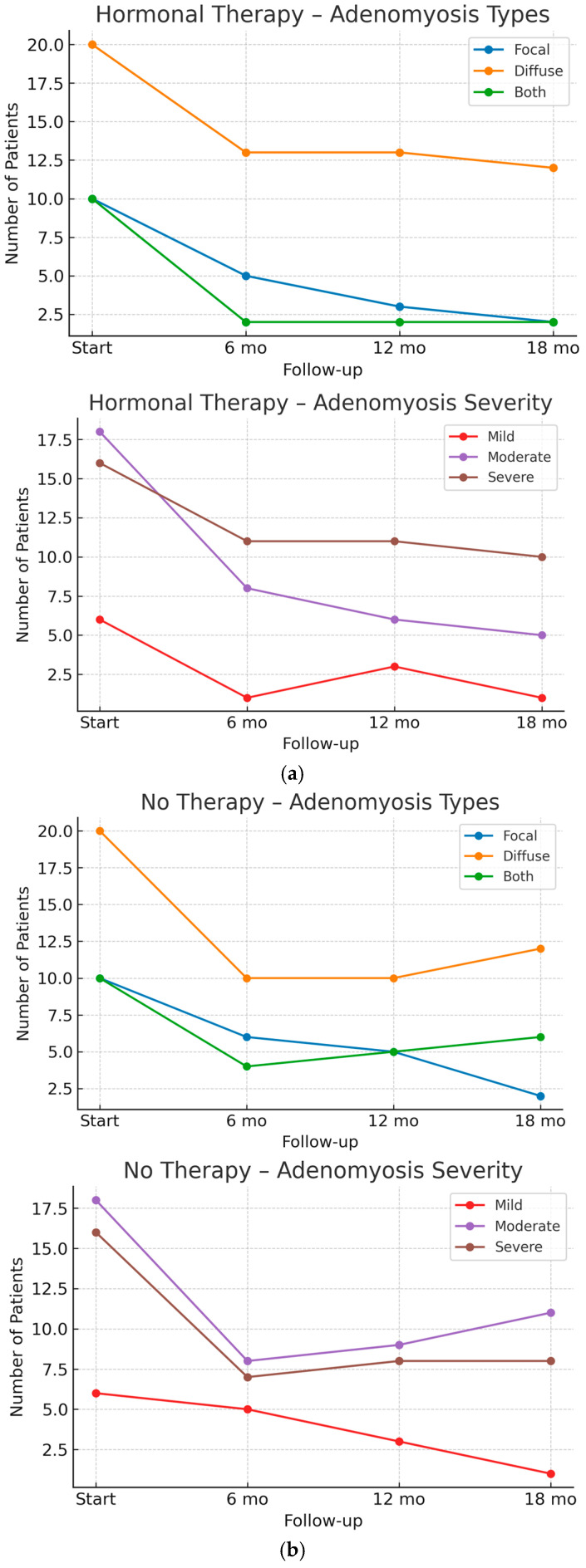
(**a**) Changes in adenomyosis ultrasound features over the 18-month follow-up in patients undergoing hormone therapy. (**b**) Changes in adenomyosis ultrasound features over the 18-month follow-up in patients without therapy.

Figure 2a,b presents the longitudinal changes in adenomyosis ultrasound features over the 18-month follow-up, distinguishing between patients undergoing hormone therapy and those not receiving treatment. The graphs illustrate the absolute number of patients diagnosed with focal, diffuse, or mixed (both) adenomyosis patterns (upper panel), as well as the distribution of severity scores classified as mild, moderate, or severe (lower panel). In the hormone-treated group, a progressive reduction in focal adenomyosis and in overall severity was observed, whereas in untreated patients, the distribution remained relatively stable or showed a mild shift toward increased severity over time.

Table 3a and b illustrate the progression of concomitant endometriosis, respectively, in patients undergoing hormonal therapy and in those not receiving any hormonal treatment.

In Table 3a, a significant reduction in the maximum diameter of endometriomas is observed at 18-month follow-up compared to baseline (T0: 38.4 ± 11.1 mm vs. T3: 18.5 ± 8.1 mm). All other endometriotic localizations remained stable in terms of maximum lesion size.

In contrast, Table 3b displays data from the 9 patients who remained without hormonal therapy. Although no statistically significant differences in lesion dimensions were detected, a trend toward increased endometrioma size was noted over time (T0: 38.4 ± 11.1 mm vs. T3: 40.2 ± 9.1 mm).

Table 4 presents the symptoms associated with adenomyosis at the time of the first ultrasound and during follow-up at 6, 12, and 18 months.

When looking at dysmenorrhea and abnormal uterine bleeding, these symptoms completely disappear in patients on continuous hormone therapy (VAS 7 ± 1.8 SD T0 vs. VAS 0 after 6 months). Conversely, in those not receiving treatment, symptoms either remain unchanged or may worsen over time.

Dyspareunia and chronic pelvic pain show a progressive reduction in patients undergoing therapy, with VAS scores decreasing to 4 after 18 months (dyspareunia VAS 7 ± 1.2 SD T0; chronic pelvic pain VAS 6 ± 1.2 SD T0). Similarly, patients on treatment report an improvement in dyschezia (VAS 6 ± 1.3 SD T0 vs. VAS 5 ± 1.2 SD after 18 months) and other previously described symptoms (VAS 6 ± 1.0 SD T0 vs. VAS 4 ± 0.8 SD after 18 months).

Dysuria decreases within the first 6 months and then stabilizes throughout the follow-up period (VAS 6 ± 1.1 SD T0 vs. VAS 4 ± 0.7 SD at 6, 12, and 18 months).

When analyzing the symptoms considered, we note that in Table 4, besides dysmenorrhea and HMB, other symptoms were not statistically significant, suggesting they may not be directly associated with the pathology studied.

Table 4 also highlights that, by the end of follow-up, nearly half of the patients undergoing hormone therapy experienced hypoestrogenism-related side effects, such as vaginal dryness, decreased libido, and mood disturbances. Specifically, 3 patients reported side effects at 6 months, 7 at 12 months, and 9 at 18 months. In contrast, no patients in the non-treatment group reported any such symptoms throughout the follow-up period, further supporting the association between hormone therapy and the development of these adverse effects.

**Figure 3:**
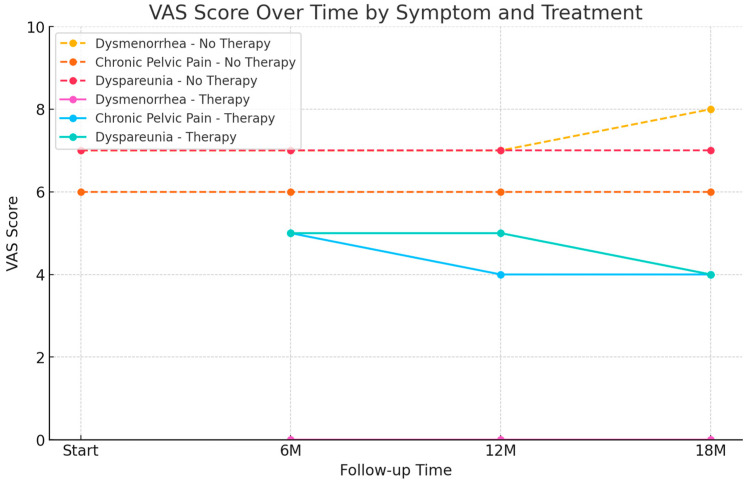
Symptom Trends in Adenomyosis With vs. Without Hormonal Therapy.

Figure 3 illustrates the evolution of VAS scores for dysmenorrhea, chronic pelvic pain, and dyspareunia in patients with adenomyosis, comparing those undergoing continuous progestin therapy and those untreated, over a follow-up period of 18 months. A clear and progressive symptom relief is observed in the treated group, with scores declining significantly as early as 6 months and maintaining improvement over time. In contrast, untreated patients show persistently high symptom levels. Notably, although not represented in the graph, HMB was completely resolved in all patients receiving continuous progestin treatment by the 6-month follow-up and remained absent throughout the entire observation period.

## 4. Discussion

Recent studies have indicated that adenomyosis typically manifests early and may progress over time in both its extent and severity [7]. This progression is often linked to an exacerbation of symptoms, which can severely impact the quality of life of affected women [7]. Unfortunately, delays in diagnosis continue to be substantial, with many women only receiving a diagnosis after years of debilitating symptoms, similar to the case of endometriosis [18]. Severe dysmenorrhea and HMB are recognized as key indicators for the risk of adenomyosis in premenopausal women [7], although these symptoms alone may not be sufficient for a definitive diagnosis [19,20]. Some studies have recommended incorporating imaging techniques, such as MRI or TVS, to non-invasively identify early signs of the disease [19,20]. Both MRI and TVS have demonstrated high accuracy in diagnosing, staging, and classifying adenomyosis [21].

The aim of our study was to explore the temporal evolution of symptom burden in patients with adenomyosis, either isolated or associated with endometriosis, with a particular focus on the clinical impact of hormonal therapy compared to no treatment, and its potential role in modulating disease progression in terms of extent and severity. We choose to evaluate the adenomyosis by ultrasound examination (TVS) which is for sure the first line diagnostic gynecological tool and that allowed to be repeated with wide availability, and low costs. TVS showed a high accuracy in diagnosing adenomyosis, with similar performance to MRI [20,21].

In our study population we observed that the prevalent symptoms were dysmenorrhea and HMB. This observation agrees with previous study [20,22]; in fact, there are no pathognomonic symptoms of adenomyosis, but dysmenorrhea and HMB are certainly the most suggestive symptoms of the pathology. The presence of other symptoms, such as dyspareunia, dysuria, dyschezia and bowel symptoms, should raise suspicion of concomitant endometriosis. Our findings support the established role of medical therapy as the first-line approach for the management of adenomyosis-related symptoms, as it appears to effectively suppress the hormonal stimulus underlying the disease. In particular, in patients with isolated adenomyosis, medical therapy often provides substantial relief from the most disabling symptoms owing to its direct action on the hormonal mechanisms driving these manifestations [23]. Conversely, in women with concomitant endometriosis, while improvements in HMB and dysmenorrhea are frequently observed, the overall benefit may be limited by the persistence of pain symptoms arising from endometriosis lesions, which are not entirely responsive to hormonal suppression [24]. These differential patterns of response highlight the importance of a personalized medicine approach, in which treatment strategies are tailored not only to the type and extent of disease but also to the individual symptom profile and patient priorities, including reproductive goals [25]. However, in the presence of concomitant endometriosis, therapeutic response may be incomplete, reflecting the multifactorial origin of pain in these patients [26]. On the other hand, in the group not on medical therapy, we had a worsening of the symptoms over time. Probably due not only to the progression of the disease, but above all to the centralization of the pain [27]. This mechanism, driven by prolonged nociceptive stimulation, may lead to altered pain processing at the central nervous system level, with persistence of symptoms even after resolution of the initial trigger [27]. Therefore, early symptom control through appropriate therapy may also represent a preventive strategy against chronic pain centralization [26,28,29].

Hence, in the presence of ultrasound findings suggestive of adenomyosis, initiation of treatment may be indicated, with medical therapy generally considered the first-line approach [30]. According to current international guidelines [31], hormonal treatments aimed at inducing amenorrhea—particularly continuous progestins or combined estrogen-progestins—are recommended as first-line options, as they are effective in alleviating symptoms such as dysmenorrhea and HMB. GnRH analogues are instead reserved as second-line therapies due to their less favorable side-effect profile and higher costs. However, their effect on other symptoms appears to vary, potentially due to the coexistence of endometriosis. While progestin therapy is highly effective in symptom control, its use is frequently limited by the onset of hypoestrogenic side effects, which may appear as early as six months into treatment and progressively worsen, affecting up to 50% of patients. In individuals whose quality of life is significantly impacted by these adverse effects, continuous estro-progestin therapy may represent a valid alternative. This approach maintains disease suppression while providing a minimal yet sufficient estrogenic component to mitigate hypoestrogenic symptoms, such as vaginal dryness, decreased libido, and mood disturbances. In patients who tolerate progestins well and do not experience significant side effects, continuation of progestin therapy remains a highly effective strategy [7]. Accordingly, in patients who do not respond adequately to continuous estro-progestin therapy, a cyclic regimen, with scheduled treatment breaks, may be considered. It is also important to consider the therapeutic option of the levonorgestrel-releasing intrauterine system (LNG-IUS), which offers a localized effect with reduced systemic side effects while maintaining effective symptom control [32].

These observations underscore the need for individualized, follow-up-based treatment plans in the management of adenomyosis. In this context, personalized medicine plays a pivotal role, enabling clinicians to adapt therapeutic regimens to the unique clinical characteristics, tolerability, and life-stage considerations of each patient. Such an approach maximizes therapeutic efficacy while minimizing side effects, ultimately improving long-term adherence and quality of life. Clinicians may alternate between progestin-only, continuous or cyclic estro-progestin regimens, and even adjust the estrogen dosage depending on patient response and tolerability [7].

In our study population we noted that the statistically significant reduction in terms of VAS of dyspareunia and dyschezia occurred only after at least 12 months of therapy, without complete regression. Specifically, these symptoms appear to be related to the presence of DE of the posterior compartment, such as USL and RVS for dyspareunia, and bowel endometriosis for dyschezia and bowel symptoms. In these locations, the presence of fibrous tissue can cause a slower and less effective reduction in the symptom [33].

In patients presenting with sonographic signs of both endometriosis and adenomyosis, medical treatment may require a longer duration to achieve efficacy, particularly for symptoms primarily attributable to the concomitant endometriosis. In such cases, a long-term therapeutic strategy is essential to enhance treatment adherence and minimize hypoestrogenic side effects, especially in younger women. The initial goal in these patients is to reduce the size of any existing endometriomas through progestin therapy, potentially achieving regression [34], thereby limiting the negative impact on the adjacent healthy ovarian parenchyma [35]. Subsequently, a continuous combined estrogen–progestin therapy with low-dose estrogen may be introduced [36,37,38].

Relative to disease progression in terms of extension and degree of adenomyosis at US scan, hormone therapy seems to be effective in reducing disease progression. Beyond symptom control, this effect may have important implications for the natural history of the disease: by slowing its evolution, medical therapy could also help preserve reproductive potential, particularly in younger patients. In this regard, timely initiation of therapy in symptomatic women—especially those with isolated adenomyosis—may represent not only a strategy for symptom relief but also an intervention aimed at limiting structural uterine damage that could compromise fertility [39]. Personalized treatment planning is therefore essential, ensuring that disease control and fertility preservation are addressed simultaneously when relevant. Adenomyosis, in fact, seems to remain unchanged in most cases and regresses in a small percentage of cases. In the group not receiving medical therapy, the disease appears to worsen in both extent and severity, with a significant number of cases progressing from focal to diffuse adenomyosis and from mild to moderate, or from moderate to severe, according to our previous classification. These findings align with two recent studies [40,41], which report disease progression over time, particularly in the absence of hormone therapy. While no studies have established a direct correlation between the degree and extent of adenomyosis and the severity of symptoms [7], our study indicates that medical therapy is effective in both alleviating symptoms and slowing disease progression. In contrast, patients not receiving medical therapy experience a deterioration of symptoms and worsening ultrasound features of adenomyosis.

Finally, it should not be forgotten that adenomyosis is very often accompanied by concomitant endometriosis [42]. This data is also confirmed in our study, as approximately 45% of patients had ultrasound signs of both pathologies. In our sample, we noted that posterior DE was more frequent in women with diffuse posterior adenomyosis, and this can be explained by the pathogenetic theory according to which adenomyosis is an expression of an external invasion of the myometrium from endometriosis [36,43]. At the same time, we observed that patients with focal adenomyosis and internal myometrial adenomyosis had less association with DE. However, it is important to evaluate the concomitant presence of endometriosis also to explain to the patient the possible persistence of symptoms, even if alleviated, despite medical therapy [44].

In young women diagnosed with adenomyosis, medical management remains the preferred first-line strategy, as it effectively controls symptoms, slows disease progression, and preserves reproductive potential. In the presence of concomitant endometriosis, surgical intervention may be considered for selected cases, particularly when symptoms are refractory to optimal hormonal therapy or when advanced lesions are compromising fertility [45]. In this age group, treatment selection should be guided by a personalized medicine approach, tailoring the therapeutic plan to the patient’s reproductive goals, symptom burden, and tolerance to medical therapy. Surgical treatment is also considered in infertility to improve chances of spontaneous conception [31]. In such cases, laparoscopy should be complemented by hysteroscopic evaluation of the uterine cavity [46]. In older women without reproductive desire, surgical management may be appropriate even in isolated adenomyosis, since hysterectomy offers definitive symptom resolution by removing the diseased uterus. However, in patients of advanced age with both adenomyosis and endometriosis, hysterectomy alone may not guarantee long-term relief, as extra-uterine endometriotic lesions can persist or progress over time, especially in advanced disease stages [33]. In such cases, a comprehensive and individualized surgical strategy that addresses both adenomyotic and endometriotic disease should be considered to maximize symptom control and improve quality of life [41,47]. Preoperative counseling remains essential, as complete symptom resolution is not always guaranteed. Additional approaches, such as dietary modifications, may also be helpful in a multidisciplinary setting [48].

Therefore, it is essential to explain to patients that the impact of medical therapy is less immediate than dysmenorrhea, in order to encourage greater adherence to therapy over time to have a more significant therapeutic benefit.

There are no established guidelines recommending specific follow-up durations to evaluate the effectiveness of medical therapy in patients with ultrasound signs of adenomyosis. However, follow-up may play a crucial role, not only in assessing disease progression but also in improving patient compliance with therapy and enhancing their quality of life.

This study was designed as a pilot exploratory analysis, intended to identify potential trends and generate hypotheses to guide the development of a larger, prospective investigation with an extended follow-up period. The limited sample size represents a key limitation, as it constrains statistical power and generalizability. Nonetheless, the consistency of our findings with existing literature supports their reliability and underscores the relevance of the observed effects. Expanding the cohort in future studies would allow for a more comprehensive evaluation of treatment outcomes, including stratified analyses by patient characteristics and a deeper assessment of long-term effects, particularly on fertility and disease progression.

Another limitation is the retrospective design of the study, which introduces potential biases and constraints in the interpretation of the results.

Another, at least possible, limitation is the absence of histopathologic diagnosis; nevertheless, the women’s age and the US advance technologies let us to make an accurate diagnosis.

A strength is, undoubtedly, the extensive experience of the ultrasound operators, as it is an Endometriosis Center, in addition to the length of the follow-up period.

## 5. Conclusions

Adenomyosis is a chronic, hormone-dependent disease that significantly impacts women during their reproductive years. This pilot study confirms the importance of medical therapy in the management of adenomyosis, supporting existing literature and highlighting its role in controlling pain symptoms such as dysmenorrhea and chronic pelvic pain. By mitigating pain, medical therapy may also reduce the risk of central sensitization, a key mechanism in chronic pelvic pain syndromes. Additionally, the reduction in disease severity observed during follow-up suggests that medical therapy may influence disease progression. This raises the hypothesis that it could also help preserve fertility, an aspect that warrants further investigation. Given the variability in clinical presentation and frequent overlap with endometriosis, management of adenomyosis requires a personalized, patient-centered approach. Tailoring treatment strategies to each patient’s symptom profile, reproductive goals, and tolerance to side effects remains essential to maximize therapeutic outcomes and improve quality of life. Although preliminary, the promising results of this pilot study justify the need for larger, long-term studies to validate these findings and further explore the therapeutic potential of medical treatment in adenomyosis.

## Figures and Tables

**Table 1:** General characteristics of the population under study, symptoms reported by the patients and ultrasound signs of adenomyosis and endometriosis.

Patients Characteristics	Total Study Population (*n* = 40) ± SD
Age	38.5 ± 4.2 SD
Body Mass Index (BMI)	20.2 ± 2.1 SD
Age at Menarche	12.6 ± 1.5 SD
Pregnancy History	0.8 ± 0.3 SD
Parity	17/40 (42.5%)
Nulliparity	23/40 (57.5%)
Symptoms	
Dysmenorrhea	32/40 (80%)
Dyspareunia	18/40 (45%)
Dysuria	7/40 (17.5%)
Dyschezia	8/40 (20%)
HMB (PBAC > 100)	30/40 (75%)
Chronic Pelvic Pain	16/40 (40%)
Other Symptoms (headaches, bowel irregularities, leukorrhea, mood changes, urinary tract infections)	9/40 (22.5%)
**Types Of Adenomyosis**	
Focal Adenomyosis	10/40 (25%)
Diffuse Adenomyosis	20/40 (50%)
Both (Focal + Diffuse Adenomyosis)	10/40 (25%)
Internal Myometrium	13/40 (32.5%)
External Myometrium	20/40 (50%)
Both (Internal + External Myometrium)	7/40 (17.5%)
Mild Degree	6/40 (15%)
Moderate Degree	18/40 (45%)
Severe Degree	16/40 (40%)
**Us Findigns Of Endometriosis**	
Endometrioma	5/40 (12.5%)
Bowel Endometriosis	4/40 (10%)
Bladder Endometriosis	2/40 (5%)
Deep Endometriosis USL	16/40 (40%) 13/40 (32.5%)
RVS	3/40 (7.5%)

HMB = heavy menstrual bleeding, PBAC: Pictorial Blood loss Analysis Chart, SD = standard deviation, RVS = rectovaginal septum, USL = uterosacral ligament.

**Table 2:** Main symptoms reported by our patients with diagnosed adenomyosis and its characteristics at baseline, i.e., at the first ultrasound examination.

Symptoms	*n*	Focal Type	Diffuse Type	Both (Focal + Diffuse Type)	Internal Myometrium	External Myometrium	Both (Internal + External Myometrium	Mild Degree	Moderate Degree	Severe Degree
Dysmenorrhea	32	9 (28.1%)	20 (62.5%)	3 (9.4%)	12 (37.5%)	18 (56.3%)	2 (6.3%)	5 (15.6%)	15 (46.9%)	12 (37.5%)
HMB (PBAC > 100)	30	10 (33.3%)	16 (53.3%)	4 (13.3%)	13 (43.3%)	10 (33.3%)	7 (23.3%)	6 (20%)	15 (50%)	9 (30%)
Dyspareunia	18	7 (38.9%)	9 (50%)	2 (11.1%)	7 (38.9%)	10 (55.6%)	1 (5.5%)	2 (11.1%)	11 (61.1%)	5 (27.8%)
Chronic Pelvic Pain	16	4 (25%)	9 (56.3%)	3 (18.7%)	6 (37.5%)	8 (50%)	2 (12.5%)	4 (25%)	6 (37.5%)	6 (37.5%)

HMB = heavy menstrual bleeding, PBAC: Pictorial Blood loss Analysis Chart.

**Table 3:** Ultrasound endometriotic findings in patients with and without hormone treatment. **3a**: Ultrasound endometriotic findings in patients at starting follow up not on hormone therapy and during hormone treatment without suspension at 6, 2 and 18 months of follow up. The largest size of endometriomas and DE lesion was considered. **3b**: Ultrasound endometriotic findings in patients not on hormone therapy at starting follow up and at 6, 12, and 18 months of follow up. The largest size of endometriomas and DE lesion was considered.

**a**								
**Ultrasound Endometriosis Findings**	**at Starting FUP** **(*n* = 18)**		**6 Months** **FUP** **(*n* = 9)**		**12 Months** **FUP** **(*n* = 9)**		**18 Months** **FUP** **(*n* = 9)**	
	**Not on Therapy**		**On Hormonal Therapy**					
	***n***	**Max Diameter (mm) Mean ± SD**	***n***	**Max Diameter (mm) Mean ± SD**	***n***	**Max Diameter (mm) Mean ± SD**	***n***	**Max Diameter (mm) Mean ± SD**
DEEP ENDOMETRIOSIS								
RVS	3	12.3 ± 3.2	2	12.1 ± 4.1	2	12.9 ± 2.6	2	12.2 ± 3.5
USL bilateral	13	7.1 ± 3.5	8	7.9 ± 2.4	8	8.1 ± 4.1	8	7.4 ± 3.2
ENDOMETRIOMA	5	38.4 ± 11.1	3	31.9 ± 10.6 *	3	20.7 ± 7.9 **	3	18.5 ± 8.1 ***
BOWEL ENDOMETRIOSIS	4	15.2 ± 3.3	1	16.1 ± NA	1	15.3 ± NA	1	15.8 ± NA
BLADDER ENDOMETRIOSIS	2	10.4 ± 2.8	1	11.2 ± NA	1	10.6 ± NA	1	10.4 ± NA
**b**								
**Ultrasound Endometriosis Findings**	**At Starting FUP (*n* = 18)**		**6 Months FUP (*n* = 9)**		**12 Months FUP (*n* = 9)**		**18 Months FUP (*n* = 9)**	
	**Not on Therapy**		**Not on Therapy**					
	***n***	**Max Diameter (mm) Mean ± SD**	***n***	**Max Diameter (mm) Mean ± SD**	***n***	**Max Diameter (mm) Mean ± SD**	***n***	**Max Diameter (mm) Mean ± SD**
DEEP ENDOMETRIOSIS								
RVS	3	12.3 ± 3.2	1	12.5 ± NA	1	12.8 ± NA	1	12.5 ± NA
USL bilateral	13	7.1 ± 3.5	5	7.3 ± 1.4	5	7.6 ± 2.8	5	7.5 ± 3.7
ENDOMETRIOMA	5	38.4 ± 11.1	2	38.9 ± 10.4	2	39.1 ± 7.6	2	40.2 ± 9.1
BOWEL ENDOMETRIOSIS	4	15.2 ± 3.3	3	15.7 ± 1.2	3	15.5 ± 2.9	3	15.4 ± 3.7
BLADDER ENDOMETRIOSIS	2	10.4 ± 2.8	1	10.8 ± NA	1	10.7 ± NA	1	10.2 ± NA

FUP = follow up, NA = not applicable, RVS = rectovaginal septum, SD = standard deviation, USL = uterosacral ligament. * *p* < 0.05 start vs. 6 months of hormonal therapy. ** *p* < 0.05 start vs. 12 months of hormonal therapy. *** *p* < 0.05 start vs. 18 months of hormonal therapy.

**Table 4:** Symptoms reported by patients with adenomyosis findings at US before and during follow up at 6, 12 and 18 months. Patients were subdivided in those who did not perform any treatment and those who undergo hormone treatment.

Symptoms								
Symptoms	At Starting (*n* = 40)		6 Months FUP (*n* = 40)		12 Months FUP (*n* = 40)		18 Months FUP (*n* = 40)	
	*n*	VAS Mean ± SD Presence *n* (%)	*n*	VAS Mean ± SD Presence *n* (%)	*n*	VAS Mean ± SD Presence *n* (%)	*n*	VAS Mean ± SD Presence *n* (%)
DYSMENORRHEA								
No Therapy	32	VAS 7 ± 1.8 SD	12	VAS 7 ± 1.8 SD	12	VAS 7 ± 1.8 SD	12	VAS 8 ± 0.7 SD
Therapy	0		20	VAS 0 *	20	VAS 0	20	VAS 0
CHRONIC PELVIC PAIN								
No Therapy	16	VAS 6 ± 1.2 SD	6	VAS 6 ± 1.2 SD	6	VAS 6 ± 1.2 SD	6	VAS 6 ± 1.2 SD
Therapy	0		10	VAS 5 ± 1.5 SD	10	VAS 4 ± 1.5 SD	10	VAS 4 ± 1.5 SD
HMB								
No Therapy	30	75%	10	100%	10	100%	10	100%
Therapy	0		20	0% *	20	0%	20	0%
DYSPAREUNIA								
No Therapy	18	VAS 7 ± 1.2 SD	6	VAS 7 ± 1.2 SD	6	VAS 7 ± 1.2 SD	6	VAS 7 ± 1.2 SD
Therapy	0		12	VAS 5 ± 0.6 SD	12	VAS 5 ± 0.6 SD	12	VAS 4 ± 0.6 SD
DYSCHEZIA								
No Therapy	8	VAS 6 ± 1.3 SD	2	VAS 6 ± 1.3 SD	2	VAS 6 ± 1.3 SD	2	VAS 6 ± 1.3 SD
Therapy	0		6	VAS 5 ± 1.2 SD	6	VAS 5 ± 1.2 SD	6	VAS 5 ± 1.2 SD
DYSURIA								
No Therapy	7	VAS 6 ± 1.1 SD	3	VAS 6 ± 1.1 SD	3	VAS 6 ± 1.1 SD	3	VAS 6 ± 1.1 SD
Therapy	0		4	VAS 4 ± 0.7 SD	4	VAS 4 ± 0.7 SD	4	VAS 4 ± 0.7 SD
OTHER SYMPTOMS								
No Therapy	9	VAS 6 ± 1.0 SD	3	VAS 6 ± 1.0 SD	3	VAS 6 ± 1.0 SD	3	VAS 6 ± 1.0 SD
Therapy	0		6	VAS 4 ± 0.8 SD	6	VAS 4 ± 0.8 SD	6	VAS 4 ± 0.8 SD
SIDE EFFECTS								
No Therapy	0	/	0	/	0	/	0	/
Therapy	0	/	3	/	7	/	9	/

FUP = follow up, US = ultrasound, HMB = heavy menstrual bleeding, SD = standard deviation; VAS: Visual Analogue Scale;. * *p* < 0.05 start vs. 6 months of hormonal therapy.

## Data Availability

The original contributions presented in this study are included in the article. Further inquiries can be directed to the corresponding author.

## Abbreviations

The following abbreviations are used in this manuscript:

BMI	Body Mass Index
1	Chronic Pelvic Pain
DE	Deep Endometriosis
FUP	Follow Up
HMB	Heavy Menstrual Bleeding
MRI	Magnetic Resonance Imaging
NA	Not Applicable
NSAID	non-steroidal anti-inflammatory drugs
PBAC	Pictorial Blood loss Analysis Chart
RVS	rectovaginal septum
SD	Standard Deviation
TVS	Transvaginal Sonography
US	Ultrasound
USL	Uterosacral ligament
VAS	Visual Analog Scale
